# A roller-like bird (Coracii) from the Early Eocene of Denmark

**DOI:** 10.1038/srep34050

**Published:** 2016-09-27

**Authors:** Estelle Bourdon, Anette V. Kristoffersen, Niels Bonde

**Affiliations:** 1 The Natural History Museum of Denmark, Section of Biosystematics, University of Copenhagen, Universitetsparken 15, DK-2100 Copenhagen, Denmark.; 2 Geological Institute (now Department of Geosciences and Natural Resource Management), University of Copenhagen, Øster Voldgade 10, DK-1350 Copenhagen, Denmark.; 3 Fur Museum (Muserum Salling), DK-7884 Fur, Denmark.

## Abstract

The fossil record of crown group birds (Neornithes) prior to the Cretaceous-Paleogene boundary is scarce and fragmentary. Early Cenozoic bird fossils are more abundant, but are typically disarticulated and/or flattened. Here we report the oldest roller (Coracii), *Septencoracias morsensis* gen. et sp. nov. (Primobucconidae), based on a new specimen from the Early Eocene (about 54 million years ago) Fur Formation of Denmark. The new fossil is a nearly complete, three-dimensionally preserved and articulated skeleton. It lies at the lower end of the size range for extant rollers. Salient diagnostic features of *Septencoracias* relative to other Coracii include the proportionally larger skull and the small, ovoid and dorsally positioned narial openings. Our discovery adds to the evidence that the Coracii had a widespread northern hemisphere distribution in the Eocene. *Septencoracias* is the oldest substantial record of the Picocoraciae and provides a reliable calibration point for molecular phylogenetic studies.

The Fur Formation of Denmark is a unique offshore marine deposit of Early Eocene age famous for its spectacular preservation of numerous fossils, including insects, fishes, turtles and birds^1^,^2^,^3^,^4^. Fossil birds from the Early Eocene Fur Formation represent the earliest post-Cretaceous bird fauna with early representatives of over 10 higher-level clades of the crown group birds (Neornithes)^3^,^5^,^6^,^7^. Sediments of the Fur Formation crop out in northwest Jutland, Denmark (supplementary Fig. S1), and consist of approximately 60 m thickness of marine diatomite interbedded with over 180 volcanic ash layers (supplementary Fig. S2)^8^. The Fur Formation is Early Eocene in age (ca. 55.8 Ma–ca. 54 Ma), based on the presence of the Paleocene/Eocene boundary in the underlying Ølst Formation^9^,^10^ and radiometric dating of two ash layers (−17 and +19) within the formation, which has yielded ages of 54.5 and 54.0 Ma^11^, the former corrected to ca. 55 Ma^12^. The sediments of the Fur Formation were deposited about 200 km off the Eocene coastline, and the depth was about 200–500 m^1^,^13^. The formation contains many groups of fishes living today at such depths or deeper^1^,^2^,^4^,^14^. Because the bottom water was probably stagnant, well-preserved, articulated vertebrate fossils are relatively common^6^,^8^,^14^. Avian remains found in the fine-grained diatomite are preserved as imprints^6^,^14^,^15^. Fossil birds from within the carbonate concretions are generally three-dimensionally preserved since the concretions protected the bones from both dissolution and compaction^8^.

Here we report a new, exquisitely preserved avian fossil from the Fur Formation. The new specimen was found in 1986 by NB and two students during a geology field course in the abandoned diatomite quarry Klovbakker on the Island of Mors^14^ (Fig. S1). The new fossil bird is preserved in a cementstone containing ash layers no. +27–+30, and it is placed immediately above ash layer +30 (an easily identified thin ‘double-layer’ with two ash falls on top of each other) (Fig. S2). The age of the fossil is approximately 54 Ma, based on (uncorrected) radiometric dating of ash layer +19, which has yielded an age of 54.04 ± 0.14 Ma^11^.

The new bird is a nearly complete, mostly articulated skeleton that is preserved uncrushed and in three dimensions, which is very rare (Fig. 1). The fossil record of crown group birds prior to the Cretaceous-Paleogene boundary is scarce and mostly consists of fragmentary remains of uncertain affinities^16^. A few sub-complete, three-dimensionally preserved neornithine fossils have been described from the Early Eocene Fur Formation of Denmark^3^,^5^,^6^,^7^,^17^ and Early Eocene Willwood Formation of Wyoming^18^,^19^,^20^. Other key deposits of Paleocene to Early Eocene age have typically yielded disarticulated or flattened specimens^16^,^21^,^22^,^23^,^24^,^25^,^26^,^27^,^28^,^29^,^30^,^31^.

The new fossil is the oldest representative of the rollers (Coracii). Crown group Coracii are a species-poor clade that comprises medium-sized birds with big heads and stout beaks^32^: the typical rollers (Coraciidae) with the Old World genera *Coracias* and *Eurystomus*, and the Madagascan ground rollers (Brachypteraciidae). Rollers generally have a brightly coloured plumage, and most species exhibit some brownish and/or bluish feathers^32^. The Paleogene fossil record of rollers consists of Eocene taxa from Europe and North America: *Geranopterus alatus* and *Geranopterus milneedwardsi* (Geranopteridae) from the Late Eocene fissure fillings of the Quercy (France)^33^; *Eocoracias brachyptera* (Eocoraciidae) from the Early Eocene deposits of Messel (Germany)^33^; *Paracoracias occidentalis* from the Early Eocene Green River Formation (Wyoming)^25^; *Primobucco mcgrewi* (Primobucconidae) from the Early Eocene Green River Formation^26^,^34^,^35^,^36^; *Primobucco frugilegus* and *Primobucco perneri* (Primobucconidae) from the Early Eocene of Messel^34^. In addition, indeterminate Geranopteridae are known from the Late Eocene Quercy deposits^33^,^37^, and a tarsometatarsus assigned to the Primobucconidae is known from the Early Eocene of Condé-en-Brie (France)^34^.

## Results

### Systematic Palaeontology

Coracii *sensu* Clarke *et al*.^25^

Primobucconidae Feduccia and Martin, 1976

*Septencoracias morsensis* gen. et sp. nov.

### Etymology

From the Latin word ‘septentrio’ meaning north, and the genus name ‘*Coracias*’. The specific epithet refers to the Island of Mors, from where the fossil bird came.

### Holotype

MGUH.VP 9509, 3-D preserved skeleton lacking sternum and most shoulder girdle elements.

### Type locality and horizon

Moclay pit (Klovbakker), Island of Mors, north-western Jutland, Denmark (Fig. S1). Fur Formation, Ypresian, Early Eocene, ca. 54 MA; marine diatomite layer right above ash layer +30 in the mid part of the formation (Fig. S2).

### Diagnosis

*Septencoracias morsensis* is a small roller that differs from all other Coracii (i.e., *Primobucco*, *Paracoracias*, *Eocoracias*, *Geranopterus* and crown group Coracii) in the small, ovoid and dorsally positioned narial openings (unknown in *Geranopterus*) and in the significantly larger skull. *Septencoracias* also differs from all Coracii except *Paracoracias* in the equal distal extent of major and minor metacarpals.

*Septencoracias* is assigned to the Primobucconidae based on the following derived characteristics: unossified nasal septum; strongly developed deltopectoral crest of humerus; alular claw present. Moreover, the morphology of the tarsometatarsus of *Septencoracias* matches well with that of the Primobucconidae. However, *Septencoracias* differs from *Primobucco* in many characters: culmen evenly curved; mandibular symphysis longer and more ventrally projected; triangular pygostyle with tall and craniocaudally narrow lamina; acromion of scapula smaller; deltopectoral crest of humerus more prominent; bicipital crest straighter and shorter in distal extent; terminal process of ischium shorter; first phalanx of hallux markedly longer.

*Septencoracias* differs from *Eocoracias* in the following characteristics: longer beak; hand skeleton longer relative to the other wing elements; femur shorter and tarsometatarsus longer relative to the tibiotarsus. *Septencoracias* differs from *Paracoracias* in the following features: extensor process of carpometacarpus larger; minor metacarpal thicker; first phalanx of hallux longer. *Septencoracias* differs from *Geranopterus* in the following characters: acromion of scapula not bifurcated; process on ventral side of proximal end of minor metacarpal smaller; plantarly projected metatarsal trochlea II.

### Description and comparison

**Figure 1:**
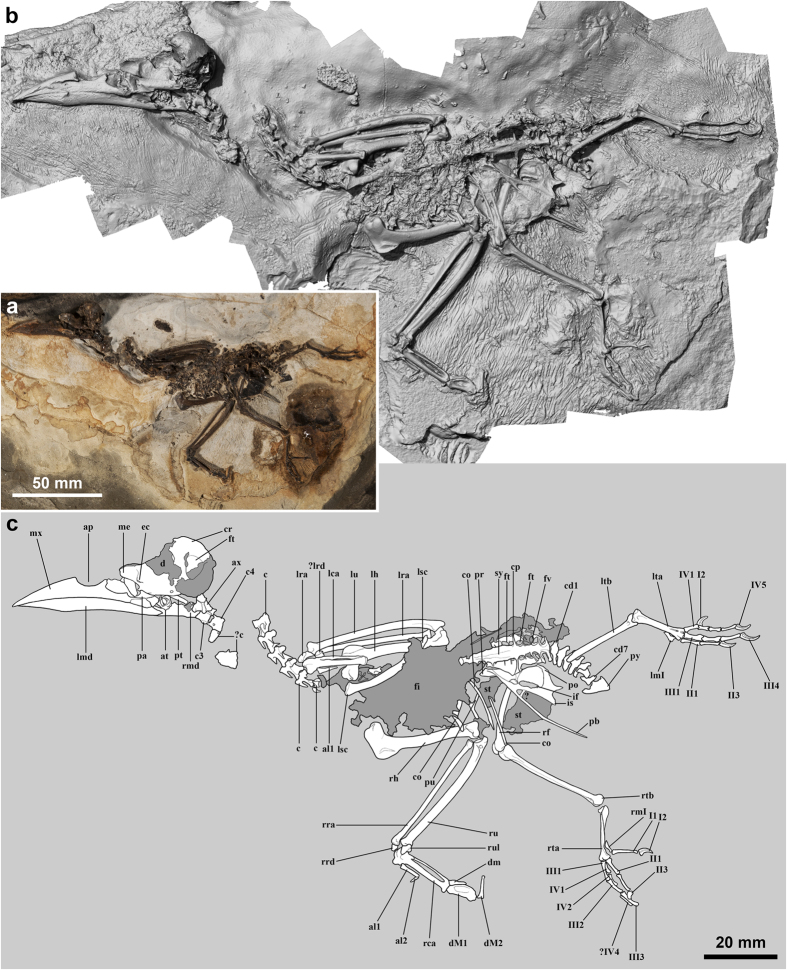
The holotype of *Septencoracias morsensis* gen. et sp. nov. (MGUH.VP 9509). (**a**) Photograph of whole specimen. (**b**) 3D white light scanning of the whole specimen. (**c**) Interpretative drawing with damaged areas of the skull, fish remains and soft tissues represented in dark grey. Abbreviations: al1–2, phalanges of alular digit; ap, narial opening; at, atlas; ax, axis; c, cervical vertebrae; ca, carpometacarpus; cd1–7, caudal vertebrae; co, vertebral costae; cp, costal process; cr, cranium; d, damage in the skull area; dm, phalanx of minor digit; dM1, proximal phalanx of major digit; dM2, distal phalanx of major digit; ec, ectethmoid; f, femur; fi, fish remains; ft, foramina intertransversaria; fv, fish vertebra; h, humerus; if, ilioischiadic foramen; is, ischium; md, mandibula; me, mesethmoid; mx, maxilla; mI, metatarsal I; pa, palatine; pb, pubis; po, postacetabular wing of ilium; pr, preacetabular wing of ilium; pt, pterygoid; pu, uncinate process; py, pygostyle; ra, radius; rd, radial carpal bone; sc, scapula; st, soft tissues; sy, synsacrum; ta, tarsometatarsus; tb, tibiotarsus; u, ulna; ul, ulnar carpal bone; I1–2, phalanges of pedal digit I; II1–3, phalanges of pedal digit II; III1–4, phalanges of pedal digit III; IV1–5, phalanges of pedal digit IV. The ‘r’ and ‘l’ prefixes indicate right and left, respectively. Artwork by E.B.

**Figure 2:**
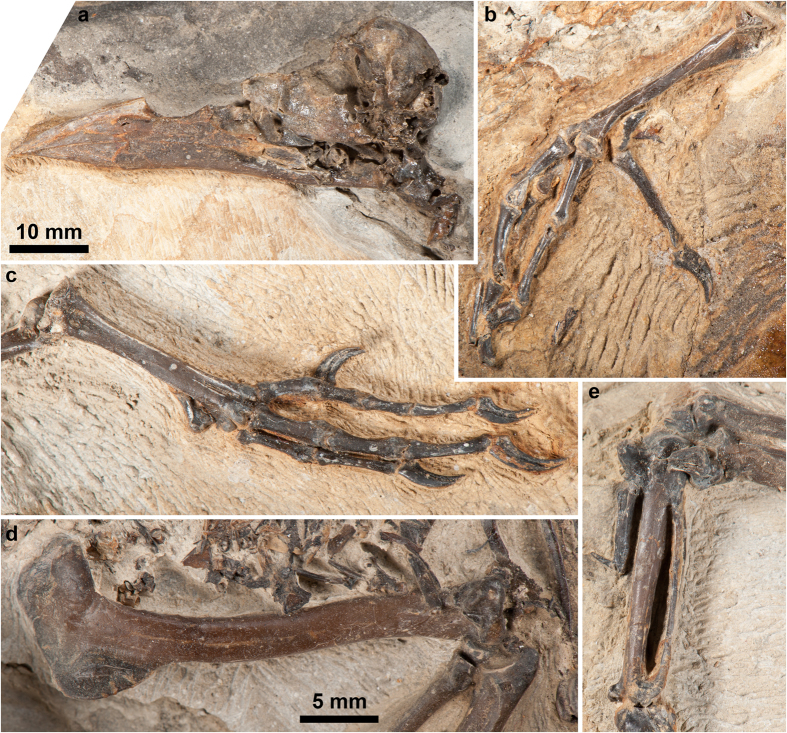
Photographs of the holotype of *Septencoracias morsensis* gen. et sp. nov. (MGUH.VP 9509). (**a**) Skull in left lateral view. (**b**) Right foot in medial view. (**c**) Left foot in dorsal view. (**d**) Right humerus in cranial view. (**e**) Right carpometacarpus in ventral view.

The skull of the new fossil is slightly eroded, because it had been exposed at the time of its discovery (Figs 1 and 2a). The head and the first five cervical vertebrae are slightly displaced from the remaining vertebral column (Figs 1 and S3). The left wing is folded tightly, whereas the right wing is partially stretched out. The right leg is still in articulation with the pelvis. Despite the bird still has the wings located close to their original position relative to the rest of the skeleton, it does not preserve the sternum, most shoulder girdle elements including the coracoids, part of the left pelvis and proximal part of the left hindlimb. Dark blotches tentatively interpreted as soft tissue remains are observable in the pelvis region (Figs 1, S3 and S4). Dark stains of carbonaceous material are also visible caudal to the right tibiotarsus (Fig. S4). These were coated with varnish prior to acid preparation and might correspond to feather remains, although poor preservation renders such an interpretation tentative. Dissociated fish remains are concentrated in the abdominal cavity and cover the thoracic vertebrae and the anterior part of the synsacrum (Figs 1, S3 and S4).

*Septencoracias* was a small bird the size of a Northern carmine bee-eater (*Merops nubicus*), and slightly smaller than the Blue-throated roller (*Eurystomus gularis*), which lies at the lower end of the size range for extant rollers and has a body length of 25 cm^32^ (Table 1). The limb elements of *Septencoracias* are roughly equal in size to those of *Primobucco frugilegus*, but its skull is much larger than that of the latter species (Table 1). *Septencoracias* is larger than *Primobucco mcgrewi* and *Primobucco perneri* and significantly smaller than other more derived fossil rollers (Table 1).

**Figure 3:**
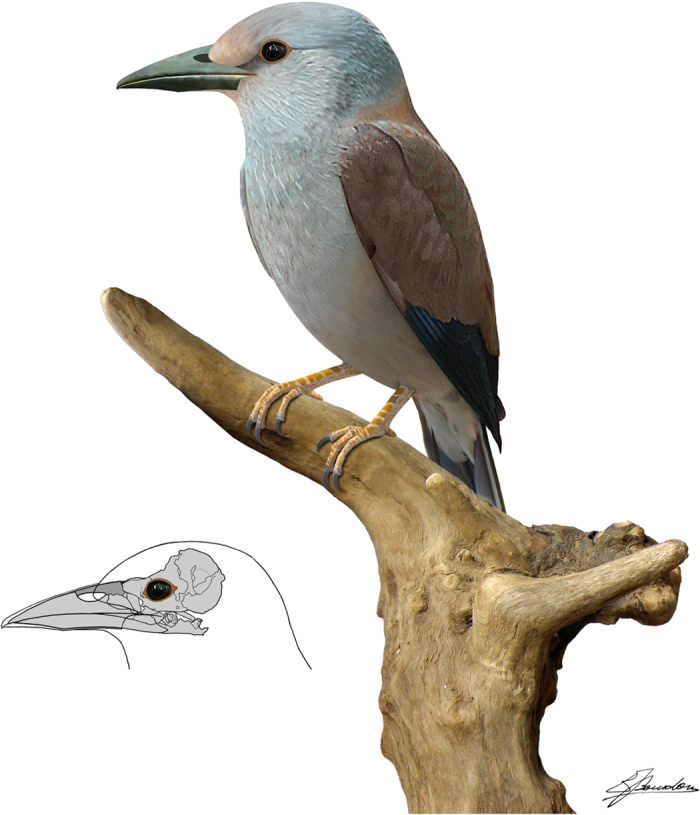
Life reconstruction of *Septencoracias morsensis* gen. et sp. nov. Salient diagnostic features of the new fossil relative to other rollers include the larger skull and the small, ovoid and dorsally positioned narial openings. *Septencoracias* is represented with a brownish and bluish plumage, because brownish and/or bluish feathers occur in all species of rollers and most species of ground-rollers^32^, and are probably primitive within the Coracii. Plumage pattern and colour are partly based on modifications from: Christian Svane (csv) - Own work, CC BY-SA 2.5, https://commons.wikimedia.org/w/index.php?curid=719240. Artwork by E.B.

The skull of *Septencoracias* is large compared to the body, being more than twice the length of the synsacrum and nearly twice the length of the humerus (Fig. 1; Tables S1 and S2). It is significantly larger than in other rollers including *Primobucco*, and is more similar in proportion to that of kingfishers, motmots, and bee-eaters (Fig. 3; Table S2). The temporal fossa of *Septencoracias* is small and shallow, unlike in extant rollers. The postorbital process is not preserved. The parasphenoid rostrum is stout and lacks basipterygoid processes. The interorbital septum is largely ossified, as in other members of the Coracii. The poorly developed ectethmoid is smaller than that of Coraciidae and does not fuse with the lacrimal or frontal. The mesethmoid is larger than in extant rollers. The lateral part of the palatine seems poorly developed, in contrast to the large lamina found in extant rollers. The right pterygoid is a stout, rod-like bone that lacks a prominent articular facet for the basipterygoid.

The beak of *Septencoracias* is stout and slightly curved. The maxillary rostrum is tall and the culmen curves gradually towards the tip of the bill, as in extant rollers (Fig. 3). In *Primobucco*, the curvature of the culmen is stronger at the anterior end. In *Septencoracias*, the length of the maxilla is slightly greater than half of the skull length (Table S2), as in *Eurystomus* (Coraciidae) and *Primobucco perneri*^34^. The maxilla of *Septencoracias* is shorter than in *Coracias* (Coraciidae) and significantly longer than in *Primobucco mcgrewi*, *Primobucco frugilegus* and *Eocoracias* (Table S2). The narial openings of *Septencoracias* are small, ovoid, dorsally positioned, and measure about ¼ of the length of the beak. They markedly differ from the elongated narial openings of *Primobucco*, the slit-like nares of *Eocoracias* and the large triangular nares of *Paracoracias* and extant rollers. The nasal septum is unossified in *Septencoracias*, in contrast to most members of the Picocoraciae including *Paracoracias* and recent rollers. The mandibular symphysis is longer than in *Primobucco*, *Eocoracias* and *Paracoracias*. It measures about ¼ of the length of the mandible and its caudal part protrudes ventrally, as in some kingfishers (Alcedinidae), e.g. *Dacelo*.

Eleven heterocoelous cervical vertebrae are observable, including the atlas and axis (Fig. 1). Seven free caudal vertebrae and the pygostyle are preserved in articulating position with the synsacrum (Figs 1 and S3). The pygostyle exhibits a lateromedially narrow caudal margin and a tall, craniocaudally narrow dorsal lamina.

The new fossil does not preserve the sternum and shoulder girdle elements, except the left scapula. The acromion of the scapula is single, in contrast to the bifurcate acromion found in Coracioidea, i.e., recent rollers and the Late Eocene *Geranopterus*^33^ (Fig. 1). Moreover, the acromion of *Septencoracias* is less prominent than in *Primobucco*. The deltopectoral crest of the humerus is well developed, as in *Primobucco*, but differs from that of the latter in the stronger development and the straight, elongated proximal margin (Figs 1 and 2d). The deltopectoral crest of the Primobucconidae is much more developed than in extant rollers, *Geranopterus* and *Eocoracias*, and somewhat larger than in *Paracoracias*. In *Septencoracias*, the deltopectoral crest is shorter than in *Primobucco mcgrewi* and extends one-quarter of the total humerus length. As in *Primobucco*, the bicipital crest is very prominent and more developed than in extant rollers. The bicipital crest of *Septencoracias* is straighter and shorter in distal extent than in *Primobucco*. The humeral shaft is slightly sigmoidal and the brachial fossa of the humerus is in median position. As in *Primobucco*, the ventral condyle of the humerus bears a shallow depression along its cranial surface.

The ulna of *Septencoracias* is longer than the humerus (Table S1). As in all other rollers, the carpometacarpus shows a ventrally protruding projection on the ventral side of the proximal end of the minor metacarpal (Fig. 1), but this projection lacks the foramen which characterizes extant rollers^33^. The extensor process is large and separated from the pisiform process by a shallow depression. The intermetacarpal space is narrow and the minor metacarpal is nearly straight. The articular surfaces of major and minor metacarpals are situated at exactly the same level (i.e. the minor metacarpal does not project distal to the major metacarpal), in contrast to *Primobucco*, in which the minor metacarpal just surpasses the major metacarpal in distal extent^25^. The carpometacarpus of *Septencoracias* and *Primobucco* lacks the prominent intermetacarpal process that is diagnostic of the Coracioidea. Manual digits are preserved on the right side (Fig. 1). *Septencoracias* shares with *Primobucco* the presence of a rudimentary claw on the phalanx of alular digit, which is absent in most higher land birds^26^,^34^. The proximal phalanx of major digit shows a deep ventral fossa and a small internal index process, as in *Primobucco*.

The wide pelvis is seen in ventral view (Figs 1 and S3). The acetabular vertebra of the synsacrum shows conspicuous costal processes, as in extant rollers. The elongated pubis is separated from the ischium by a large ischiopubic fenestra. The left femur is not preserved, and the right one is still in articulation with the pelvis. Hind limb proportions are roughly similar to those of typical rollers (Coraciidae), *Primobucco* and *Paracoracias* (Table S2). The tarsometatarsus is very short, i.e., it measures about half the length of the tibiotarsus, as in *Eurystomus*, *Primobucco mcgrewi* and *Paracoracias* (Fig. 1; Table S2). However, the tarsometatarsus of *Septencoracias* is less abbreviated than in kingfishers (Alcedinidae) and bee-eaters (Meropidae), in which this bone measures less than half the length of tibiotarsus (Table S2). As in other members of the traditional ‘Coraciiformes’, the tarsometatarsus of *Septencoracias* shows a deep medial parahypotarsal fossa, with a very sharp proximal part of medial margin of shaft (Fig. 2b); another ‘coraciiform’ feature of *Septencoracias* is the metatarsal trochlea IV reaching almost as far distally as trochlea III and being rotund in lateral view (Fig. 2c). As in other Coracii, the metatarsal trochleae are arranged on a convex line in distal view. The metatarsal trochlea II is plantarly projected, in contrast to the condition found in the Coracioidea. The tarsometatarsus of *Septencoracias* exhibits the features of the Primobucconidae^34^, including: tarsometatarsal shaft slender in its midsection and widening towards proximal and distal ends; distal part of medial margin of tarsometatarsus forming sharp oblique ridge; tuberositas musculi tibialis cranialis tubercle-like and situated towards the medial margin of the shaft; medianoplantar crest prominent and bordered by deep medial parahypotarsal fossa; large distal vascular foramen located at the end of a marked sulcus. Our phylogenetic analysis shows that some of these features actually characterize the whole Coracii and therefore are primitive within the roller group (see discussion below).

The foot of *Septencoracias* has an anisodactyl toe arrangement. The first phalanx of pedal digit (PD) I is markedly longer than the first phalanx of PDIII (Figs 1 and 2b), as opposed to *Primobucco*, in which ph1PDI is subequal in length to ph1PDIII (Table S2). The elongated ph1PDI approaches the condition found in *Eurystomus*. However the relative lengths of PDI and PDIII of *Septencoracias* are close to those of other rollers (Table S2). *Septencoracias* lacks the medial expansion on the proximal end of ph1PDI that characterizes the Alcediniformes^34^,^38^. PDIV is subequal in length to PDIII and much longer than PDII, as in *Primobucco mcgrewi*, *Paracoracias* and recent rollers (Figs 1 and 2).

## Discussion

Phylogenetic analysis based on morphological data indicates that *Septencoracias* is sister taxon to *Primobucco*^26^,^34^ and that the Primobucconidae are stem group representatives of the Coracii (Fig. S5). *Geranopterus*^33^, *Paracoracias*^25^ and *Eocoracias*^33^ are successive sister taxa of crown group Coracii (represented here by *Coracias* and *Atelornis*). In agreement with recent molecular and morphological studies, we provide evidence that the Coracii are nested within the clade Picocoraciae^39^, which also includes the Alcediniformes (kingfishers, bee-eaters, motmots, todies^38^), Bucerotiformes (hornbills), Upupiformes (hoopoes) and Piciformes (woodpeckers, barbets and puffbirds)^25^,^40^,^41^.

*Septencoracias* constitutes the oldest record of the Primobucconidae and the earliest occurrence of the Coracii. It indicates that the Coracii were already diversified by the earliest Eocene, some 54 MA. The second oldest record of that clade consists of a tarsometatarsus from the Early Eocene (MP8-9, ca. 53–52 MA) of Condé-en-Brie^42^, which also belongs to the Primobucconidae^34^. As such, the new fossil provides a reliable calibration point for molecular phylogenetic studies. *Septencoracias* unambiguously shares with *Primobucco* an unossified nasal septum (Fig. 2a); a very prominent deltopectoral crest of humerus (Fig. 2d) and an alular claw (Fig. 1). Placement of *Septencoracias* within the rollers (Coracii) is strongly supported. *Septencoracias* unambiguously shares with extant and extinct rollers the derived presence of a small depression on the cranial face of humeral head (Fig. 1b); a narrow and elongate brachial fossa of the humerus; a ventrally protruding projection on the ventral side of the proximal end of minor metacarpal (Fig. 1); a deep dorsal infracotylar fossa on the tarsometatarsus (Figs 1 and S3); a sharp distal part of medial margin of tarsometatarsal shaft (Fig. 2b); a greatly enlarged distal vascular foramen of tarsometatarsus prolonged by a marked groove (Fig. 2c).

*Septencoracias* lacks the large triangular narial openings with flat ventral margin that characterize the clade including Coracioidea (extant rollers plus *Geranopterus*) and *Paracoracias*. These triangular narial openings are associated with a rhamphotheca forming slit-like nostrils in extant rollers and ground-rollers^32^. *Septencoracias* is not represented with slit-like nostrils in our life reconstruction, since its osseous nasal apertures strongly differ in shape and position to those of extant rollers (Fig. 3). *Septencoracias* lacks the prominent intermetacarpal process and the distally projecting minor metacarpal that characterize the Coracioidea. In addition, *Septencoracias* lacks derived features of crown group Coracii (Coraciidae and Brachypteraciidae), including: large temporal fossae approaching each other at midline; transverse sulcus of humerus deep and bipartite; presence of foramen on the ventral process of proximal end of minor metacarpal.

*Septencoracias* constitutes the oldest substantial record of the Picocoraciae, since the only other contemporaneous record of that clade is only tentative^23^. The syndactyl foot is optimized here as a derived feature of the Picocoraciae including fossil rollers, and secondarily lost in piciform birds, which exhibit a zygodactyl foot instead (PDIV oriented backwards). The syndactyl foot occurs in rollers, kingfishers, bee-eaters, motmots, todies, hoopoes and hornbills^32^. In these birds, PDIII and PDIV are coalescent at least over length of proximal phalanx of PDIII. In the right foot of *Septencoracias*, PDIII and PDIV lie on top of each other, with PDII separated from these (Fig. 2b), which might suggest syndactyly. However, syndactyly cannot be ascertained in *Septencoracias*, because PDIV is slightly displaced from PDIII in the left foot (Fig. 2c).

The fish remains located in the thoracic and abdominal regions of *Septencoracias* are from the most common fish in the Fur Formation, a small argentinoid^1^,^4^,^14^ of which adult individuals reach approximately 10 cm in length. This small ‘argentine’ constituted the main food source for many contemporary sea predators^4^. In the abdominal cavity of the bird, remains of at least two individuals are recognizable, one adult and one juvenile. These fish remains are tentatively interpreted as preserved stomach content, rather than animals fossilized on top of each other. Fish remains may have been dissociated owing to partial digestion, and possibly spread outside the abdominal cavity owing to decay of soft tissues and/or mechanical breakage. Our interpretation concurs with the hypothesis that stem rollers had more flexible foraging habits than living species of rollers^33^,^34^. However, since *Septencoracias* is a land bird, it is probable that its diet included arthropods and small terrestrial vertebrates, like Crown group Coracii^32^.

Extant rollers are restricted to the Old World, and all species occur in tropical to subtropical regions, except the European roller *Coracias garrulus* and the Dollarbird *Eurystomus orientalis*, which are also recorded in the temperate zone^32^. The Fur Formation was deposited just after the Paleocene-Eocene Thermal Maximum^9^, and the Fur Formation fauna and flora indicate a tropical to subtropical environment in the earliest Eocene^4^. The fauna and flora of the Green River Formation also indicate a tropical to subtropical climate in the Early Eocene^43^. Our discovery provides further evidence that the Coracii had a widespread northern hemisphere distribution in the Eocene, with subsequent restriction of the clade to the Old World tropics and subtropics^25^. This is consistent with a pattern found in numerous clades of crown group birds, in which taxa now restricted to low latitude tropical environments were present in higher latitudes in the Eocene^16^,^44^.

## Methods

### Taxonomy

We use the name Coracii sensu Clarke *et al*.^25^ to designate the clade including extant rollers (Coraciidae and Brachypteraciidae) and their extinct relatives. The term Alcediniformes sensu Mayr^38^ is used to designate the clade including Alcedinidae, Meropidae, Momotidae and Todidae. The name Picocoraciae sensu Mayr^39^ is used to designate the clade including Piciformes, Coracii, Alcediniformes, Bucerotiformes and Upupiformes. The traditional ‘Coraciiformes’ is an informal term used to designate the members of the clade Picocoraciae, except the Piciformes. The name Coraciimorphae designates the clade including Piciformes, Coracii, Alcediniformes, Bucerotiformes, Upupiformes, Trogoniformes, Leptosomiformes and Coliiformes^40^,^41^.

### 3D white light scanning

3D scanning was made with a Breuckmann stereoSCAN 3D White Light Scanner. The point accuracy is 0,007 mm for a single scan. There are 1.400.000 points in a single scan, and the resolution is 710 dpi. The object was scanned from many angles, and has a total of 13.239.516 points in the file. The scanner software OPTOCAT was used for 3D image processing.

### Phylogenetic analysis

Phylogenetic analysis is based on a morphological dataset of 21 taxa and 78 characters. Sixty characters (1 to 60) were taken from previous phylogenetic analyses^25^,^34^. Eighteen characters (61 to 78) are new. Taxon sampling in the ingroup includes seven taxa of the Coracii (5 Eocene fossils plus 2 extant genera), 5 extant genera of the Alcediniformes, 1 extant genus of the Bucerotiformes, 2 extant genera of the Upupiformes and 3 extant genera of the Piciformes. Three outgroups were included to root trees: the extant genera *Tyto*, *Colius* and *Harpactes*. *Colius* and *Harpactes* were already used in the original dataset of Clarke *et al*.^25^. We replaced *Caprimulgus* by *Tyto* in our analysis, because recent molecular studies indicate that Strigiformes are the sister group of the Coraciimorphae^40^,^41^. Extant taxa were scored from skeletons deposited in the collections of the Muséum National d’Histoire Naturelle in Paris (MNHN), the Natural History Museum in Tring (NHMUK), and the Natural History Museum of Denmark (SNM). The parsimony analysis was performed using PAUP v4b10^45^. All characters were unordered, except multistate characters 13 and 17, which were treated as ordered. The branch-and-bound algorithm was used for the PAUP program. The phylogenetic analysis yielded one single most parsimonious tree (length = 204 steps, consistency index = 0.42 and retention index = 0.65).

## Additional Information

**How to cite this article**: Bourdon, E. *et al*. A roller-like bird (Coracii) from the Early Eocene of Denmark. *Sci. Rep*. **6**, 34050; doi: 10.1038/srep34050 (2016).

## Supplementary Material

Supplementary Information

## Figures and Tables

**Table 1:** Measurements (mm) in *Septencoracias morsensis* gen. et sp. nov. (MGUH.VP 9509), other representatives of the Coracii and *Merops nubicus* (Alcediniformes).

	SK	RM	SC	HU	UL	CM
*Septencoracias morsensis* HO	57.3	30.7	28.0	31.6	37.8	17.6
*Primobucco mcgrewi*^26^,^34^	40.5	—	21–23.3	26.7–28	32.5–34.2	14.2–15.7
*Primobucco perneri*^34^	38.6–43	—	—	25.2–29.3	32–36.3	15–17.1
*Primobucco frugilegus*^34^	46.6–48	—	—	31.5–32.7	37.8–38.4	18.7–19.4
*Eocoracias brachyptera* HO^33^	58	—	—	45	54.3	25.0
*Paracoracias occidentalis* HO^25^	60.0	30.6	36.0	43.7	52.7	25.9
*Geranopterus alatus*^33^	—	—	—	47.7	—	26.1
*Eurystomus glaucurus* MNHN-LAC 2008–13	52.5	28.0	34.0	47.3	57.4	26.6
*Eurystomus gularis* MNHN-LAC 1880–119	55.5	29.5	35.0	51.0	63.0	28.7
*Coracias caudatus* ZMUC 08.06.1999–51	—	—	32.7	49.0	56.3	—
*Coracias abyssinica* MNHN-LAC 1854–195	—	—	31.5	45.5	55.0	24.5
*Coracias garrulus* MNHN-LAC 1997–1080	61.0	37.0	30.0	47.1	55.7	25.8
*Coracias benghalensis* MNHN-LAC 1997–919	66.7	39.7	33.4	53.0	64.3	27.5
*Coracias naevius* ZMUC 18.12.1998–22	—	—	37.0	54.2	—	—
*Merops nubicus* ZMUC 25.01.2013–6	59.3	38.6	27.5	35.0	45.1	19.8
	**SY**	**FM**	**TT**	**TM**	**BL**	
*Septencoracias morsensis* HO	25.5	20.5	30.9	15.5	—	
*Primobucco mcgrewi*^34^	—	19	26.7	13.1	—	
*Primobucco perneri*^34^	—	18.4	20.9–24	11.5–13.1	—	
*Primobucco frugilegus*^34^	—	—	—	14	—	
*Eocoracias brachyptera* HO^33^	—	32.1	41.3	18.2	—	
*Paracoracias occidentalis* HO^25^	—	29.4	39.7	19.2	—	
*Geranopterus alatus*^33^	—	—	—	—	—	
*Eurystomus glaucurus* MNHN-LAC 2008–13	25.2	26.0	33.2	17.3	270–290	
*Eurystomus gularis* MNHN-LAC 1880–119	27.1	27.6	36.7	18.5	250	
*Coracias caudatus* ZMUC 08.06.1999–51	25.5	29.0	—	—	280–300	
*Coracias abyssinica* MNHN-LAC 1854–195	24.5	27.7	39.0	21.7	280–310	
*Coracias garrulus* MNHN-LAC 1997–1080	26.5	26.8	39.2	22.2	310–320	
*Coracias benghalensis* MNHN-LAC 1997–919	31.3	32.0	46.0	25.7	300–340	
*Coracias naevius* ZMUC 18.12.1998–22	30.5	33.0	—	—	350–400	
*Merops nubicus* ZMUC 25.01.2013–6	24.5	19.0	26.8	12.3	240–270	

Abbreviations: BL, body length; CM, carpometacarpus; FM, femur; HO, holotype; HU, humerus; MNHN-LAC, Muséum National d’Histoire Naturelle, Laboratoire d’Anatomie Comparée (Paris); RM, maxillary rostrum; SC, scapula; SK, skull; SY, synsacrum; TM, tarsometatarsus; TT, tibiotarsus; UL, ulna; ZMUC, Zoological Museum, Copenhagen University.
