# Supplementary material for: A roller-like bird (Coracii) from the Early Eocene of Denmark
Source: Sci Rep. 2016 Sep 27;6:34050. doi: 10.1038/srep34050 (PMC5037458; doi:10.1038/srep34050)
Supplement: Supplementary Information [file srep34050-s1.pdf]

A roller-like bird (Coracii) from the Early Eocene of Denmark

Estelle Bourdon, Anette V. Kristoffersen & Niels Bonde

Supplementary text

1. Character/taxon matrix

	000000000111111111122222222223333333333
	123456789012345678901234567890123456789
Tyto	0000001100001000000000000000001000010?00
Colius	100000100001000000001000010100000010?01
Harpactes	000000100000002100000100000000000000?01
Septencoracias	?00?000????0????0??00???00????000001000
Primobucco	10??0001?0??101?0??1?000???000000001000
Eocoracias	?0???0?100??201?0???00?0??00?0001001?00
Paracoracias	11??001100??201?0??10?????0?0000000??00
Geranopterus	??0?????????21??????????1000??0??01101?
Coracias	111101111010211010010100110000000011110
Atelornis	111100111000211000010100100000001111110
Baryphthengus	00010010?100101111000001100100001010?10
Todus	1001001010000011110000011?0100101010?10
Merops	001000000000001110000001110010001011010
Alcedo	001110111010000110000001101000101010?10
Dacelo	001110111010101110000001100000001010?10
Upupa	1001111??1?1001020101010011011111120?11
Phoeniculus	1001111??1?1000020101011011011111120?11
Bucorvus	1001011000?1101020001010010001101020?10
Nystalus	111100110011201010101210001000001110?10
Pteroglossus	0200011??1?1101020001021010100112010?10
Picoides	0000000??1?110101?001021110100112110?10

	444444444455555555556666666666777777777
	012345678901234567890123456789012345678
Tyto	001000000000000001000000000001001000000
Colius	00000000010000001000?0100001000000000010
Harpactes	000000001000000000000100000001000001001
Septencoracias	001000?0??111?0100???00100?011011100111
Primobucco	001000????11?101?0???1???0?0?1011100011
Eocoracias	?00001????11??01?0???0???00??10???00000
Paracoracias	?00001????1???01?0???10??0????01?1?0101
Geranopterus	0??00?????1?1101???????100101101110??01
Coracias	000001000011110100100101101011011100101
Atelornis	00000000001111010010?101101101010100100
Baryphthengus	000000000011000110111100010000000010100
Todus	01000000001100011011110001010?000?10100
Merops	000000000011000110101000000101001011100
Alcedo	000001000011100110101101010101001011100
Dacelo	000000000011100110101100010101011011100
Upupa	110011101101000101100010000100100010000
Phoeniculus	110011101111000111100010000100100010100
Bucorvus	000000101011000100100010010000100010000
Nystalus	00010000001100100000001001010?100000100
Pteroglossus	000100010010002000000010110100100010100
Picoides	000100010100002001000111110100100010100

2. Character list (new characters marked with an asterisk)

1. Relationship of narial opening and antorbital fenestra: do not overlap in lateral view (0); narial opening overlaps antorbital fenestra posteriorly (1)[1: ch1].
2. Narial opening, shape, lateral view: ovoid (0); triangular with a flat ventral margin (1); not exposed, small, round and dorsally positioned (2)[1: ch2].
3. Temporal fossae, dorsal extent: widely separated (0); approach each other at midline (1)[1: ch3].
4. Quadrate with strongly ventrally protruding medial condyle: no (0), yes (1)[2: ch8].
5. Palatine, caudolateral angle, posteriorly directed spine-like process: absent (0); present (1)[1: ch4].
6. Narial opening, division by thin bony septum: undivided (0); divided (1)[1: ch6].
7. Nasal septum, largely or completely ossified: absent (0); present (1)[1: ch7,2: ch1].
8. Lacrimal, head: small (0); greatly enlarged (1)[1: ch10,2: ch3].
9. Lacrimal, orbital process: unexpanded (0); greatly medially expanded (1)[1: ch11,2: ch4].
10. Lacrimal: present, unreduced (0); greatly reduced or absent (1)[1: ch12].
11. Lacrimal, posterior margin of head in dorsal view: straight (0); concave, with supraorbital process (1)[1: ch13 modified].
12. Ectethmoid, greatly expanded and plate-like, dorsal margin fused with frontals: no (0); yes (1)[1: ch14].
13. Postorbital process: short (0); elongate, but well separated from jugal bar (1); elongate, touching or nearly touching jugal bar (2)[1: ch15]. Ordered.
14. Postorbital process, development of an anterior process: absent (0); present (1)[1: ch16,2: ch6].
15. Zygomatic process, weak or absent: (0); long and thin (1); abbreviated and tab-like (2)[1: ch17].
16. Columella, large hollow bulbous basal foot plate area with large fenestra on one side: absent (0); present (1)[1: ch19,2: ch7,3].
17. Mandibular symphysis: short, less than one-third of mandible length (0); more than one-third of mandible length (1); extensive, at least half of mandible length (2)[1: ch20 modified]. Ordered.
18. Mandible, deep incision between medial process and retroarticular process in dorsal view: absent (0); present (1)[1: ch21,2: ch10].
19. Presacral vertebrae: more than 18 (0); 18(1)[1: ch26 modified].
20. Pygostyle, anterior border of lamina: notch absent (0); notch present (1)[1: ch27 modified].
21. Pygostyle, discus: unmodified (0); shield-like with sharply defined ridge-like lateral margins (1)[1: ch28 modified,2: ch11].
22. Furcula, apophysis: absent or small, blunt tubercle (0); blade-like projection (1); large sheet-like expansion (2)[1: ch29].
23. Furcula, omal end: unmodified (0); widened with blunt, slightly convex and short acrocoracoid and acromial processes (1); acrocoracoid and acromial processes well developed and wide, forming plate-like omal extremity of subtriangular shape (2)[1: ch30].
24. Furcula, shaft of clavicle very narrow at sternal end: no (0), yes (1)[2: ch12].
25. Scapula, acromion: single (0); bifurcate, with additional medial process (1)[1: ch31,2: ch14].
26. Scapula, pneumatic foramen on anterior part of acromion: absent (0); present (1)[1: ch32].
27. Coracoid, bony bridge connects procoracoid process and acrocoracoid process: absent (0); present (1)[1: ch33].
28. Coracoid, procoracoid process: well developed (0); greatly reduced (1)[1: ch34,2: ch15].
29. Sternum, internal spine: absent (0); present (1)[1: ch37].

30. Sternum, caudal incisures: four (0); two (1)[1: ch40].
31. Ulna, remigal papillae: absent or faint (0); prominent raised knobs (1)[1: ch43].
32. Ulna, olecranon: blunt (0); elongated and narrow (1)[1: ch44].
33. Ulnare, rami: ventral ramus (crus longus) longer than dorsal ramus (crus brevis) (0); subequal (1); dorsal ramus longer than ventral ramus (2)[1: ch45].
34. Carpometacarpus, major metacarpal, anterior metacarpal protuberance at midshaft: absent (0); present (1)[1: ch46].
35. Carpometacarpus, intermetacarpal process: absent or weak (0); well developed (1); absent, but tendon of m. extensor carpi ulnaris inserting in position of intermetacarpal process (2)[1: ch47,2: ch19].
36. Carpometacarpus, minor metacarpal with ventrally protruding projection on ventral side of proximal end: absent (0); present (1)[1: ch48,2: ch20].
37. Carpometacarpus, foramen in ventrally protruding projection from minor metacarpal: absent (0); present (1)[1: ch49]. This character is considered non comparable for taxa lacking the projection.
38. Carpometacarpus: major and minor metacarpals subequal in length (0); minor metacarpal projects significantly distal to major metacarpal (1)[1: ch50,2: ch22].
39. Carpometacarpus, major and minor metacarpals: separated by moderate intermetacarpal space (0); minor metacarpal strongly bowed, creating a wide space (1)[1: ch52].
40. Carpometacarpus, ridge continuing distally from pisiform process to minor metacarpal; absent (0); present (1)[1: ch53].
41. Carpometacarpus, posterior margin of minor metacarpal: smooth (0); undulating (1)[1: ch54].
42. Phalanx of alular digit, rudimentary claw: absent (0); present (1)[2: ch24,4,5].
43. Proximal phalanx of major digit, large proximally directed process on ventral side: absent (0); present (1)[1: ch55,2: ch23].
44. Proximal phalanx of major digit, proximally hooked process projects from caudal edge of distal end: absent (0); present (1)[1: ch56].
45. Proximal phalanx of major digit, internal index process: small, does not surpass the phalangeal articular face (0); well developed, surpasses phalangeal articular face (1)[1: ch57].
46. Femur, pneumatic foramen on anterolateral surface of proximal end: absent (0); present (1)[1: ch60,2: ch25].
47. Tibiotarsus, cranial cnemial crest continuous with ridge along medial edge of shaft, paralleling the fibular crest: absent (0); present (1)[1: ch62].
48. Tarsometatarsus, bony canal enclosing tendons of m. flexor hallucis longus: absent (0); present (1)[1: ch63,2: ch28].
49. Tarsometatarsus, ossified extensor retinaculum: absent (0); present (1)[1: ch64].
50. Tarsometatarsus with very marked medial parahypotarsal fossa, proximal part of medial margin forming sharp ridge: no (0), yes (1)[2: ch27].
51. Tarsometatarsus, well-developed medianoplantar crest: absent (0); present (1)[1: ch66,2: ch26].
52. Tarsometatarsus, distal vascular foramen: moderate size (0); greatly enlarged (1)[1: ch67,2: ch29].
53. Tarsometatarsus, distal interosseal canal: ossified (0); plantarly not ossified, forming deep, narrow groove on the plantar surface of the bone, between trochleae III and IV (1)[1: ch68,2: ch30].

54. Tarsometatarsus, large accessory trochlea on trochlea IV: absent (0); present, abbreviate (1); present, extending distally to be subequal in distal extent with trochlea III (2)[1: ch69].
55. Tarsometatarsus trochlea IV reaching almost as far distally as trochlea III and rotund in lateral view: absent (0); present (1)[1: ch70,2: ch32].
56. Pedal digit I, phalanx 1, proximal end: unexpanded (0); greatly medially expanded (1)[1: ch71,2: ch33].
57. Pedal digit I, phalanx 1, length relative to other proximal phalanges: moderate length, e.g. subequal to phalanx III:1 (0); elongate, twice the length of phalanx III:1 (1)[1: ch72].
58. Foot syndactyl, i.e. pedal digits III and IV coalescent at least over length of proximal phalanx of pedal digit III: no (0); yes (1)[1: ch73,2: ch34].
59. Maxilla, cutting edge of rhamphotheca finely serrated: absent (0); present (1)[1: ch9,2: ch2].
60. Musculus flexor hallucis longus: tendon supplying hallux (0); tendon not supplying hallux (1)[1: ch129,2: ch36,6].
61. Neurocranium: shorter than or subequal to synsacrum in length (0); largely exceeding synsacrum in length (1).*
62. Synsacrum, costal process of acetabular vertebra: well developed (1); reduced to absent (0).*
63. Humerus: small depression in ventral part of cranial face of humeral head: absent (0); present (1).*
64. Humerus, transverse sulcus: shallow (0); deep and bipartite, sharply delimiting caput (1).*
65. Humerus, deltopectoral crest: well developed, triangular in shape (0); reduced, distal part of crest straight and elongate (1).*
66. Humerus, caudal face of proximal end, thin longitudinal crest on median axis of bone, turning at right angle to reach base of dorsal margin of pneumotricipital fossa: absent (0); present (1)[7: 539].*
67. Humerus, brachial fossa: median position (0); extreme ventral position (1).*
68. Humerus, brachial fossa: wide and/or short proximodistally (0); narrow and elongate (1).*
69. Humerus, shallow depression along cranial surface of ventral condyle: absent (0); present (1).*
70. Proximal phalanx of major digit: ovoid, with convex caudal margin (0); rectilinear, with straight caudal margin (1).*
71. Tarsometatarsus, dorsal infracotylar fossa: shallow (0); deep (1).*
72. Tarsometatarsus, distal part of medial margin: blunt (0); forming sharp, oblique ridge (1).*
73. Tarsometatarsus, marked groove proximal to distal vascular foramen: absent (0); present (1).*
74. Tarsometarsus, trochleae: arranged on a convex line in distal view (0); on the same dorsoplantar level, so that dorsal plane above trochleae flat (2).*
75. Tarsometatarsus: measuring half of more than half the length of tibiotarsus (0); greatly abbreviated, measuring less than half the length of tibiotarsus (1).*
76. Pedal digit IV: shorter than pedal digit III and subequal to pedal digit II (0); subequal to pedal digit III in length (1).*
77. Humerus, deltopectoral crest: moderately developed (0); very prominent (1).*
78. Humerus, bicipital crest: moderately developed, merges smoothly into the shaft (0); very prominent, angles sharply into the shaft (1).*

3. List of unambiguous synapomorphies (homoplastic ones marked with an asterisk)

Node A: 13(0)*; 39(1)*; **Node B**: 4(1); 15(1); 50(1); 51(1); 55(1); 58(1); **node C**: 35(0)*, 36(1)*, 52(1)*, 53(1), 63(1)*, 69(1)*, 72(1)*, 73(1); **node D**: 13(2)*, 45(1)*; **node E**: 2(1)*, 61(1)*; **node F**: 14(1), 35(1)*, 38(1)*; **node G**: 3(1)*, 37(1), 64(1)*; **node H**: 17(1)*, 33(1)*, 38(1)*, 65(1), 67(1)*, 74(1); **node I**: 16(1)*, 24(1)*, 25(1)*, 56(1)*, 60(1); **node J**: 18(1), 28(1)*, 59(1); **node K**: 3(1)*, 69(1)*, 72(1)*, 75(1)*; **node L**: 5(1)*, 11(1)*, 52(1)*; **node M**: 12(1)*, 21(1)*, 23(1), 62(1)*, 70(1); **node N**: 6(1)*, 17(2)*, 30(1)*, 35(2), 46(1), 48(1)*; **node O**: 5(1)*, 10(1)*, 13(0)*, 19(1)*, 27(1)*, 29(1)*, 32(1)*, 39(1)*, 40(1), 41(1)*, 44(1), 45(1)*, 49(1)*, 57(1)*, 65(0)*; **node P**: 43(1), 55(0)*, 58(0)*; **node Q**: 4(0)*, 10(1)*, 23(2), 24(1)*, 28(1)*, 32(1)*, 33(2), 47(1), 51(0)*, 64(1)*; **node R**: 7(0)*, 42(1)*, 77(1)*.

Supplementary figures

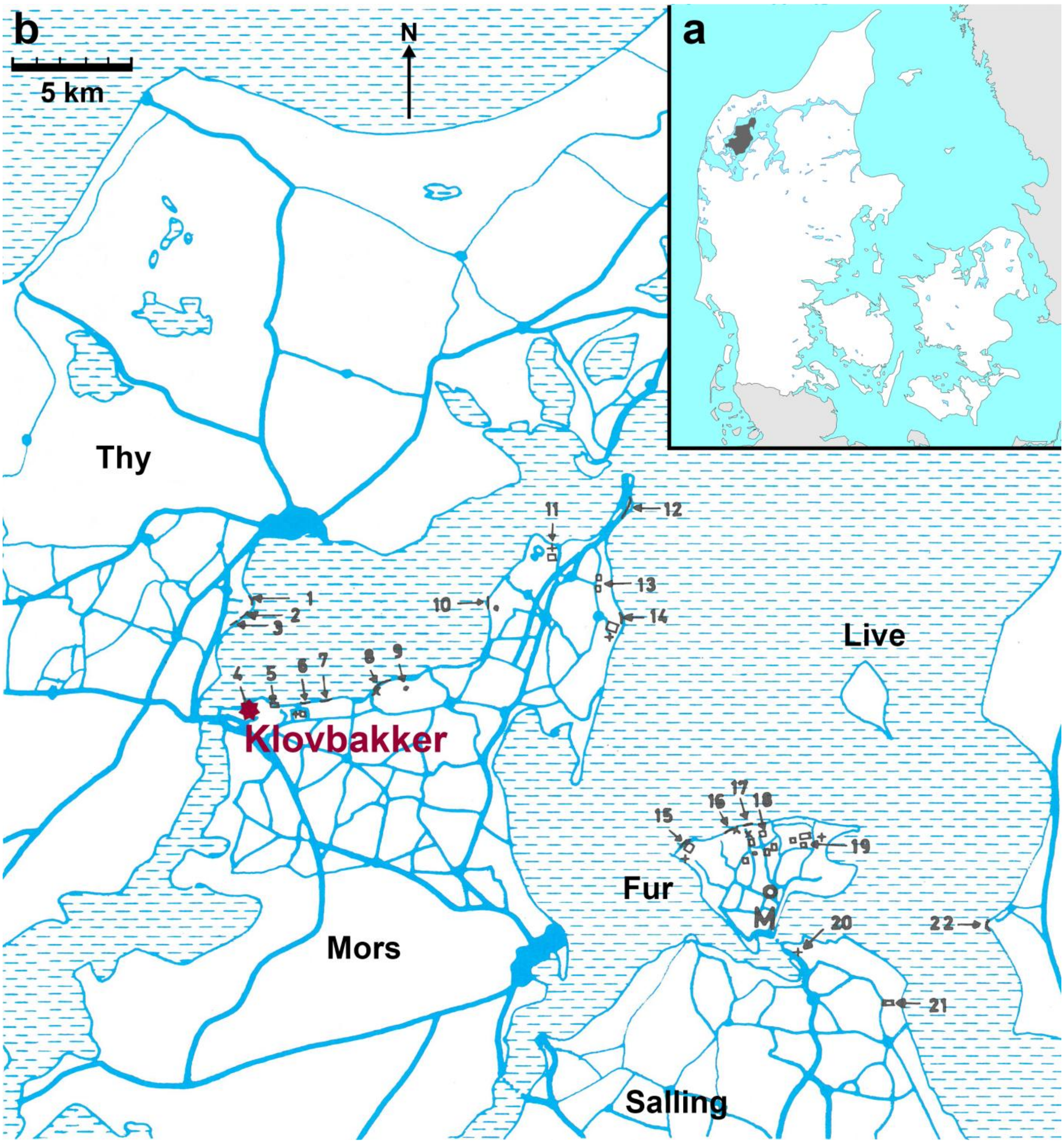

Figure S1. Locality of the new species. (**a**) Map of Denmark, with Island of Mors in dark grey; modified from d-maps.com (http://d-maps.com/m/europa/danemark/danemark13.pdf). (**b**) Map of northwest Jutland, Denmark, showing the localities of the Fur Formation (numbers); modified from Bonde[8]. The red star indicates the locality Klovbakker, from where the fossil bird came. Image generated with Adobe Photoshop CS6 (http://www.adobe.com).

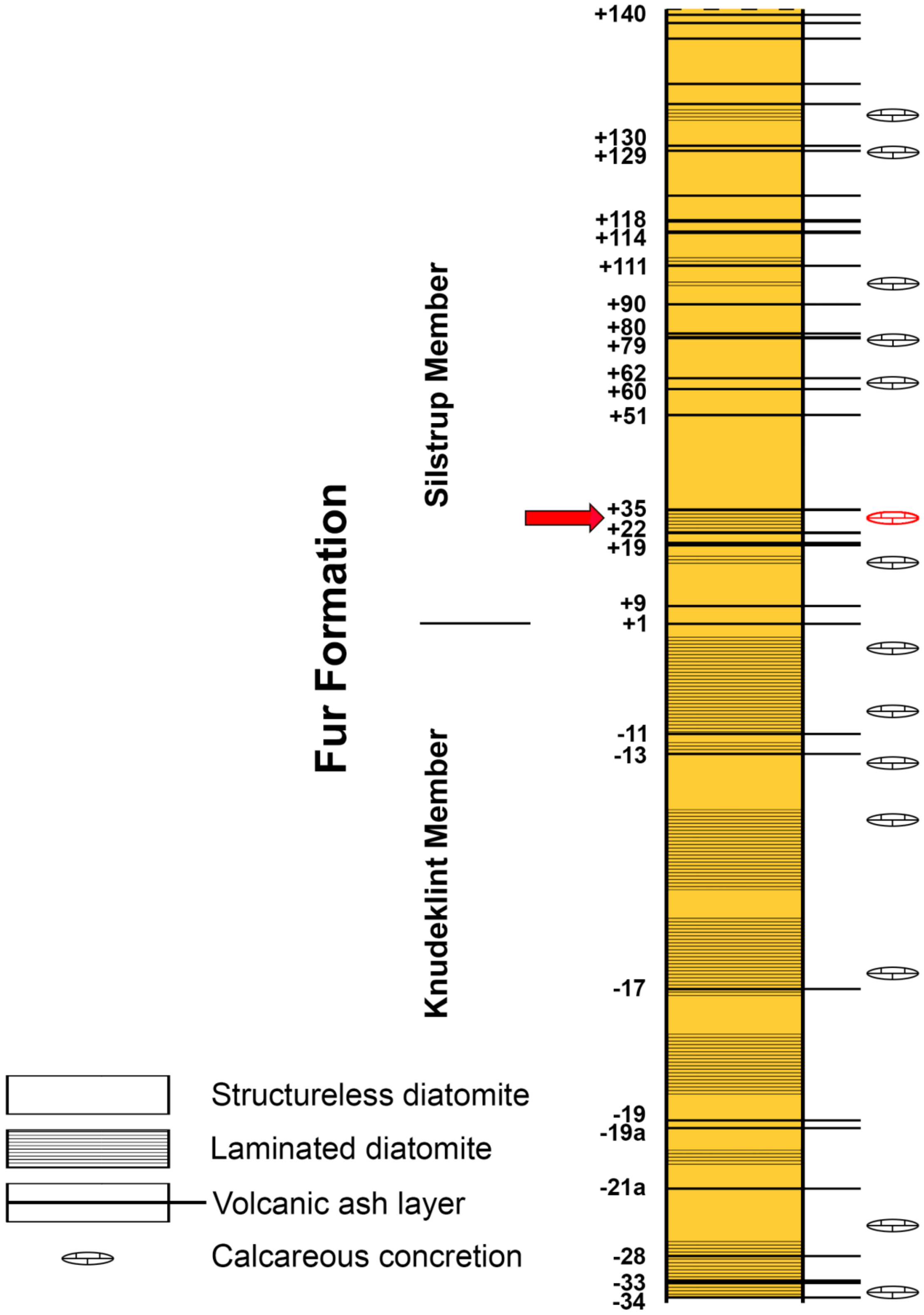

Figure S2. Horizon of the new species. Stratigraphic section of the Fur Formation showing the ash layers, the horizons containing calcareous concretions and the provenance of the new fossil bird (red arrow).

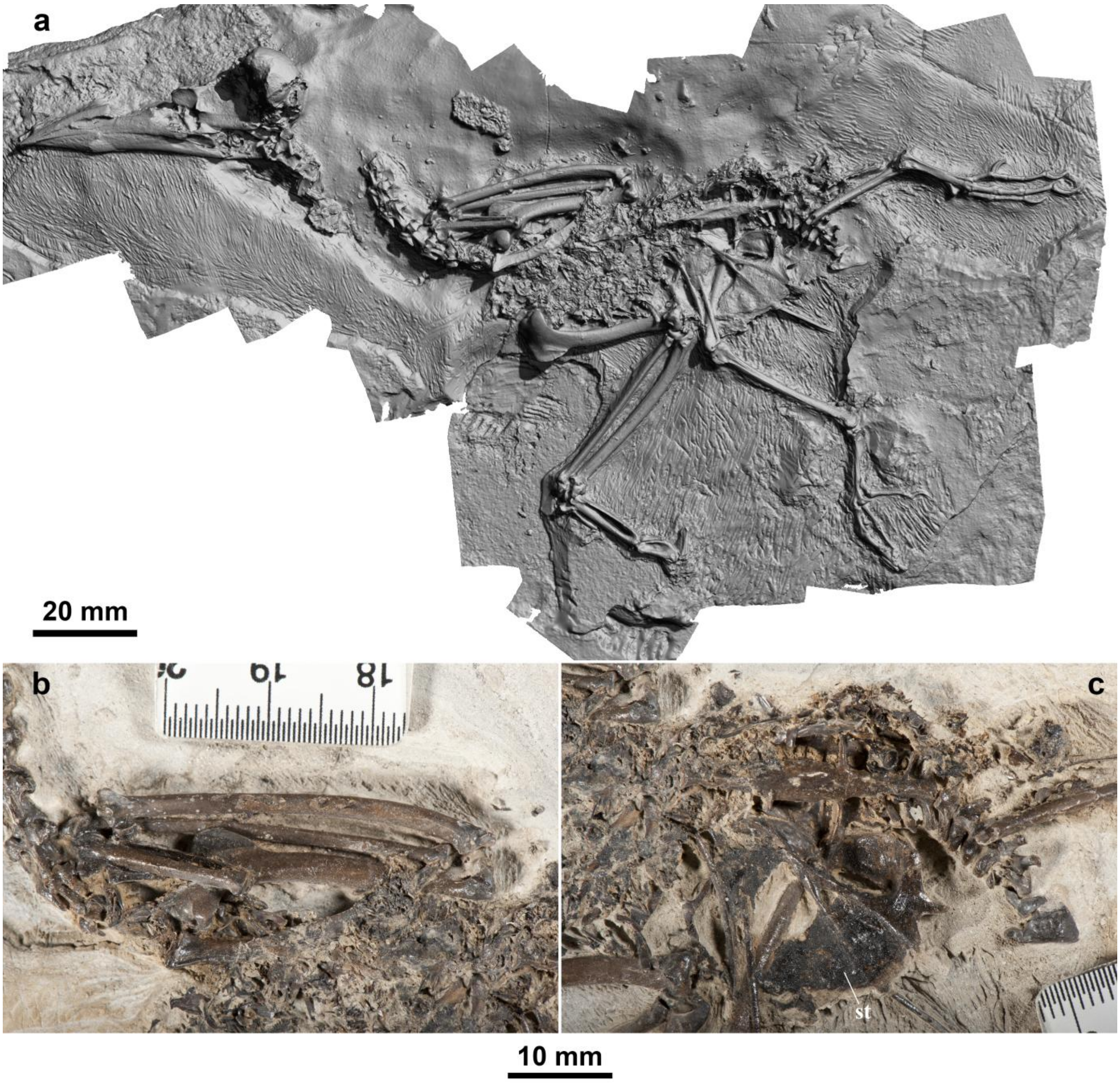

Figure S3. The holotype of *Septencoracias morsensis* gen. et sp. nov. (MGUH.VP 9509). (**a**) 3D white light scanning of the whole specimen. (**b**) Left wing and fish remains. (**c**) Pelvis and fish remains. Abbreviations: st, soft tissues.

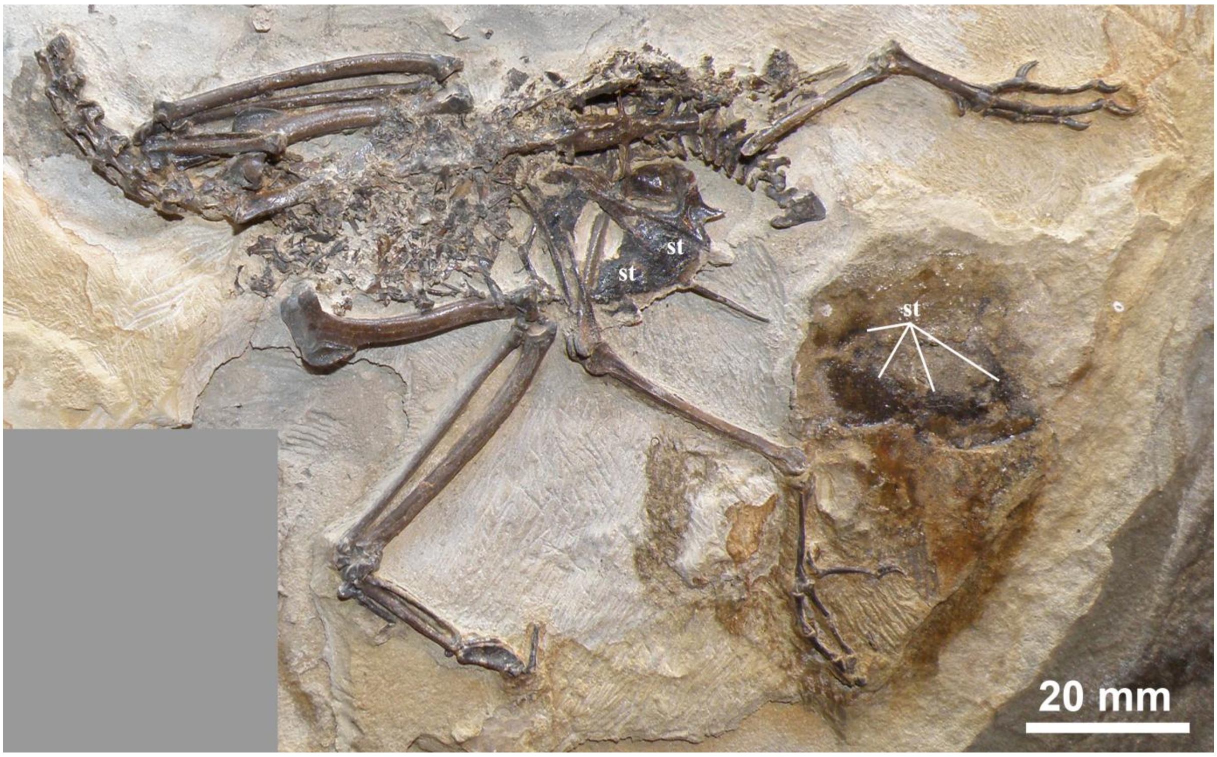

Figure S4. Photograph of the holotype of *Septencoracias morsensis* gen. et sp. nov. (MGUH.VP 9509), postcranial skeleton. Abbreviations: st, soft tissues. Photograph by E.B.

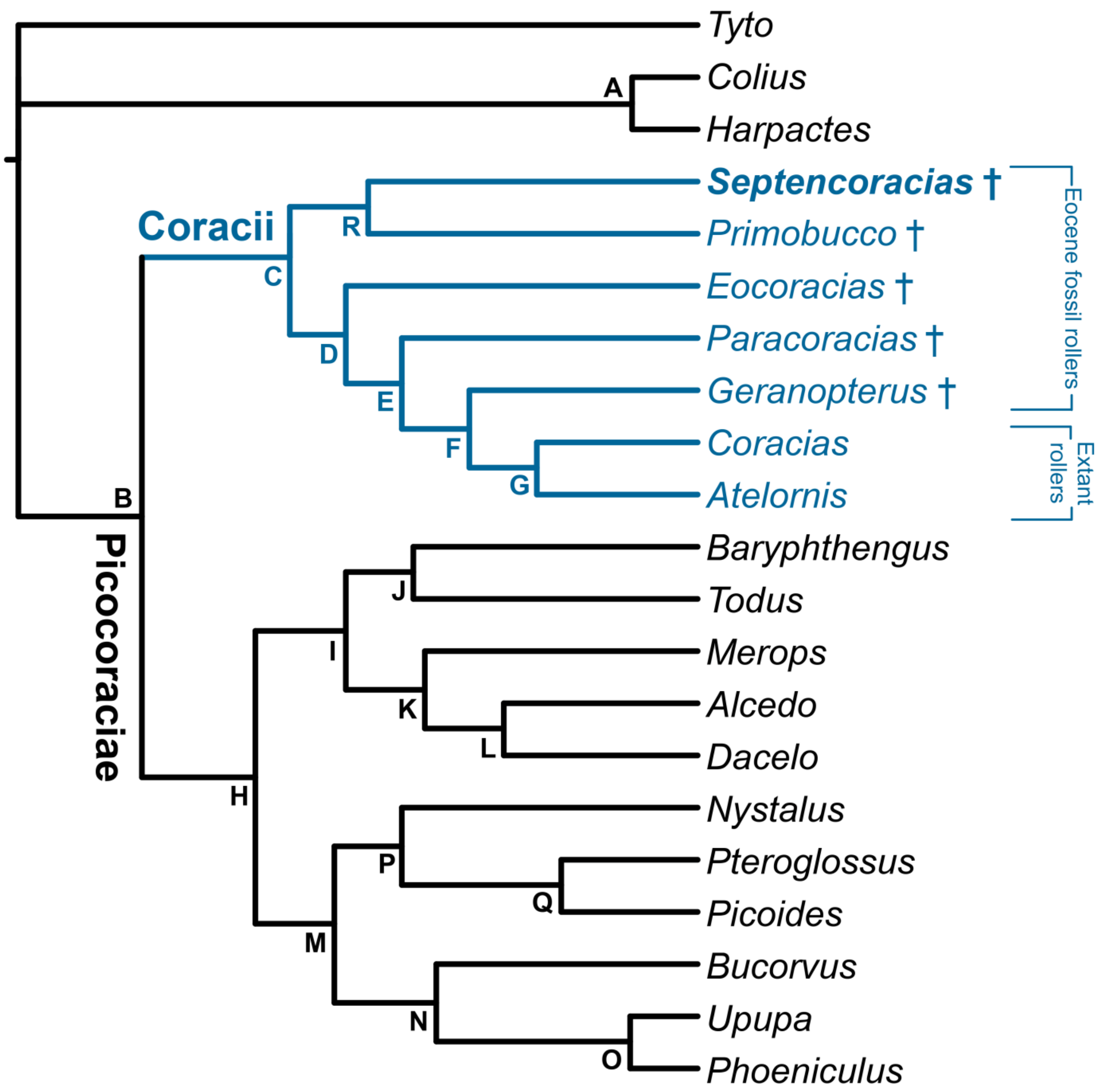

Figure S5. Phylogenetic position of *Septencoracias morsensis* with respect to other members of the Picocoraciae. Single most parsimonious trees derived from phylogenetic analysis based on 21 taxa and 78 morphological characters. Tree length = 204, consistency index = 0.42, retention index = 0.65. The Primobucconidae are placed at the base of the Coracii.

Supplementary tables

Measurement	Value
Skull, total length	57.3
Braincase, length	27.0
Braincase, height	16.0*
Maxillary rostrum, length	30.7*
Maxillary rostrum, length from tip to rostral end of narial opening	18.3
Narial opening, minimal length	9.0
Mandibula, length	48.5
Axis, length	5.3*
Axis, width	5.9
Synsacrum, length	25.5
Synsacrum, maximum width	12.7*
Pygostyle, height	7.3
Scapula, length (left)	28.0
Humerus, length (right/left)	31.6
Humerus, maximal width of proximal end	10.1
Humerus, length of pectoral crest	8.0*
Humerus, length from caput to distal end of pectoral crest	8.9
Humerus, length from distal extremity to distal end of pectoral crest	22.7
Humerus, width of shaft at mid-height	3.2
Ulna, length (right/left)	37.4/38.2 (mean 37.8)
Radius, length (right)	35.5
Carpometacarpus, length (right/left)	17.2/18.0 (mean 17.6)
Phalanx 1 alular digit, length (right)	6
Phalanx 2 alular digit, length (right)	1.7
Phalanx 1 of major digit, length (right)	8.4
Phalanx 2 of major digit, length (right)	6.3
Phalanx of minor digit, length (right)	4.9
Wing skeleton, length (right)	100*
Renal fossa, length	15.0
Shaft of pubis, length	22.8
Femur, length (right)	20.5
Tibiotarsus, length (right)	30.9
Tibiotarsus, width of shaft	1.7
Tibiotarsus, distal width (left)	3.5
Tarsometatarsus, length (right/left)	15.5
Tarsometatarsus, proximal width	4.1
Tarsometatarsus, minimal width of shaft	1.7
Tarsometatarsus, distal width	3.7
Tarsometatarsus, proximal depth	3.6
First metatarsal, length	3.4
Pedal digit I (right): ph1/ph2	8.3/4.1
Pedal digit II (left): ph1/ph2/ph3	5.4/5.1/4.6
Pedal digit III (left): ph1/ph2/ph3/ph4	5.4/5.2/5.7/5.2
Pedal digit IV (left): ph1/ph2/ph3/ph4/ph5	4.2/3.7/3.5/4.2/4.0

Table S1. Measurements of *Septencoracias morsensis* gen. et sp. nov. (MGUH.VP 9509) in mm. * indicates estimated value.

	SK/RM	SK/HU	SK/CM	SK/SY	SK/TM	HU/CM
Septencoracias morsensis HO	1.86	1.81	3.25	2.24	3.69	1.79
Primobucco mcgrewi USNM336284[2]	2.20*	1.47	2.64	-	3.09	1.79
Primobucco perneri HO[2]	1.89*	1.43	2.75	-	3.2	1.92
Primobucco frugilegus SMF ME 3794[2]	2.16*	1.46	2.47	-	3.42	1.68
Eocoracias brachyptera HO[7]	2.04*	1.28	2.32	-	3.18	1.80
Paracoracias occidentalis HO[1]	1.96	1.37	2.31	-	3.12	1.68
Eurystomus glaucurus MNHN-LAC 2008-13	1.87	1.10	1.97	2.08	3.03	1.77
Eurystomus gularis MNHN-LAC 1880-119	1.88	1.08	1.93	2.04	3	1.77
Coracias abyssinica MNHN-LAC 1854-195	-	-	-	-	-	1.85
Coracias garrulus MNHN-LAC 1997-1080	1.64	1.29	2.36	2.30	2.74	1.82
Coracias benghalensis MNHN-LAC 1997-919	1.68	1.25	2.42	2.13	2.59	1.92
Quasisyndactylus longibrachis HO/SMFME 3543[9,10]	-/1.80*	-/2.24	-/4.08	-/-	-/3.62	2.12/1.81
Dacelo gigas ZMUC 13.10.1983	1.71	1.50	2.93	2.76	3.98	1.94
Merops nubicus ZMUC 25.01.2013-6	1.53	1.69	2.99	2.42	4.82	1.76
Baryphthengus ruficapillus ZMUC 4.8.41	1.84	1.58	3.17	2.62	2.52	2
Trogon surrucura ZMUC 9.3.1849.8	2.51	1.31	2.24	2.18	2.82	1.7
	HU/TM	FM/TM	TT/FM	TT/TM	Ph1DI/ Ph1DIII	PDI/ PDIII
Septencoracias morsensis HO	2.03	1.32	1.51	1.99	1.53	0.57
Primobucco mcgrewi USNM336284[2]	2.09	1.45	1.40	2.03	0.95*	0.45*
Primobucco perneri HO[2]	2.23	1.40	1.30	1.83	1.0*	-
Primobucco frugilegus SMF ME 3794[2]	2.33	-	-	-	1.04*	-
Eocoracias brachyptera HO[7]	2.47	1.76	1.29	2.26	1.24	0.45
Paracoracias occidentalis HO[1]	2.27	1.53	1.35	2.06	1.22	0.55
Eurystomus glaucurus MNHN-LAC 2008-13	2.73	1.50	1.28	1.91	-	-
Eurystomus gularis MNHN-LAC 1880-119	2.75	1.49	1.33	1.98	1.66	0.60
Coracias abyssinica MNHN-LAC 1854-195	2.09	1.27	1.41	1.79	1.23	0.52
Coracias garrulus MNHN-LAC 1997-1080	2.12	1.20	1.46	1.76	-	-
Coracias benghalensis MNHN-LAC 1997-919	2.06	1.24	1.44	1.78	-	-
Quasisyndactylus longibrachis HO/SMFME 3543 [9,10]	2.06/1.61	1.38/-	1.42/-	2.04/1.76	1.5/-	-
Dacelo gigas ZMUC 13.10.1983	2.64	1.5	1.53	2.28	0.97	-
Merops nubicus ZMUC 25.01.2013-6	2.84	1.54	1.41	2.17	-	-
Baryphthengus ruficapillus ZMUC 4.8.41	1.59	1.12	1.52	1.71	1.08*	-
Trogon surrucura ZMUC 9.3.1849.8	2.14	1.47	1.40	2	1*	0.42*

Table S2. Proportions of bones in *Septencoracias morsensis* gen. et sp. nov. (MGUH.VP 9509), other Coracii, Alcediniformes and *Trogon surrucura*. E, estimated ratio. Abbreviations: CM, carpometacarpus; FM, femur; HO, holotype; HU, humerus; RM, maxillary rostrum; SK, skull; SY, synsacrum; TM, tarsometatarsus; TT, tibiotarsus. * indicates estimated ratio.

Supplementary references

1 Clarke, J. A., Ksepka, D. T., Smith, N. A. & Norell, M. A. Combined phylogenetic analysis of a new North American fossil species confirms widespread Eocene distribution for stem rollers (Aves, Coracii). *Zool. J. Linn. Soc.* **157**, 586–611 (2009).

2 Mayr, G., Mourer-Chauviré, C. & Weidig, I. Osteology and systematic position of the Eocene Primobucconidae (Aves, Coraciiformes sensu stricto), with first records from Europe *J. Syst. Palaeontol.* **2**, 1–12 (2004).

3 Feduccia, A. Morphology of the bony stapes (columella) in the Passeriformes and related groups: evolutionary implications. *Univ. Kansas Mus. Nat. Hist. Misc. Publ.* **63**, 1–34 (1975).

4 Ksepka, D. T. & Clarke, J. A. *Primobucco mcgrewi* (Aves: Coracii) from the Eocene Green River Formation: new anatomical data from the earliest constrained record of stem rollers. *J. Vert. Paleontol.* **30**, 215–225 (2010).

5 Stephan, B. Vorkommen und Ausbildung der Fingerkrallen bei rezenten Vögeln. *J. Ornithol.* **133**, 251–277 (1992).

6 Maurer, D. A. & Raikow, R. J. Appendicular myology, phylogeny, and classification of the avian order Coraciiformes (including Trogoniformes). *Ann. Carnegie Mus.* **50**, 417–434 (1981).

7 Mayr, G. & Mourer-Chauviré, C. Rollers (Aves: Coraciiformes s.s.) from the Middle Eocene of Messel (Germany) and the Upper Eocene of the Quercy (France). *J. Vert. Paleontol.* **20**, 533–546 (2000).

8 Bonde, N. Det jyske moler. *Varv* **2**, 44–55 (1972).

9 Mayr, G. "Coraciiforme" und "piciforme" Kleinvögel aus dem Mittel-Eozän der Grube Messel (Hessen, Deutschland). *Cour. Forsch.-Inst. Senckenberg* **205**, 1–101 (1998).

10 Mayr, G. New specimens of *Hassiavis laticauda* (Aves: Cypselomorphae) and *Quasisyndactylus longibrachis* (Aves: Alcediniformes) from the Middle Eocene of Messel, Germany. *Cour. Forsch.-Inst. Senckenberg* **252**, 23–28 (2004).
